# Establishing seasonal and alert influenza thresholds in Morocco

**DOI:** 10.1186/s12889-020-09145-y

**Published:** 2020-06-29

**Authors:** Ahmed Rguig, Imad Cherkaoui, Margaret McCarron, Hicham Oumzil, Soumia Triki, Houria Elmbarki, Abderrahman Bimouhen, Fatima El Falaki, Zakia Regragui, Hassan Ihazmad, Chakib Nejjari, Mohammed Youbi

**Affiliations:** 1 Direction of Epidemiology and Disease Control, MoH, Rabat, Morocco; 2 Centers for Disease Control and Prevention, Atlanta, USA; 3 National Institute of Hygiène, NIC, MoH, Rabat, Morocco; 4 WHO Office of Morocco, Rabat, Morocco; 5 University Mohammed VI of Health Sciences, Casablanca, Morocco

**Keywords:** Influenza seasonality, Average epidemic curve, Seasonal threshold, Alert threshold

## Abstract

**Background:**

Several statistical methods of variable complexity have been developed to establish thresholds for influenza activity that may be used to inform public health guidance. We compared the results of two methods and explored how they worked to characterize the 2018 influenza season performance–2018 season.

**Methods:**

Historical data from the 2005/2006 to 2016/2018 influenza season performance seasons were provided by a network of 412 primary health centers in charge of influenza like illness (ILI) sentinel surveillance. We used the WHO averages and the moving epidemic method (MEM) to evaluate the proportion of ILI visits among all outpatient consultations (ILI%) as a proxy for influenza activity. We also used the MEM method to evaluate three seasons of composite data (ILI% multiplied by percent of ILI with laboratory-confirmed influenza) as recommended by WHO.

**Results:**

The WHO method estimated the seasonal ILI% threshold at 0.9%. The annual epidemic period began on average at week 46 and lasted an average of 18 weeks. The MEM model estimated the epidemic threshold (corresponding to the WHO seasonal threshold) at 1.5% of ILI visits among all outpatient consultations. The annual epidemic period began on week 49 and lasted on average 14 weeks. Intensity thresholds were similar using both methods. When using the composite measure, the MEM method showed a clearer estimate of the beginning of the influenza epidemic, which was coincident with a sharp increase in confirmed ILI cases.

**Conclusions:**

We found that the threshold methodology presented in the WHO manual is simple to implement and easy to adopt for use by the Moroccan influenza surveillance system. The MEM method is more statistically sophisticated and may allow a better detection of the start of seasonal epidemics. Incorporation of virologic data into the composite parameter as recommended by WHO has the potential to increase the accuracy of seasonal threshold estimation.

## Background

Seasonal influenza epidemics result in considerable annual morbidity and mortality, with an estimated 291,243 to 645,832 deaths per year globally [1]. Associated with these seasonal epidemics are substantial economic losses due to absenteeism, lost wages and increased utilization of health care services [2]. The influenza-associated respiratory annual mortality rate for people aged 65 and older in Morocco has been recently estimated by the US Centers for Disease Control and Prevention (US CDC) at 3.7 per 100,000 (95% Credible Interval of 0.4–22.3) [1]. The risk of hospitalization due to influenza is 5 to 10 times greater in high-risk populations in Morocco (e.g., the elderly and people with chronic disease) than in the general population [3]. The most effective ways to prevent or mitigate these effects are through vaccination combined with appropriate clinical management of persons infected with influenza. Optimal impact of vaccination campaigns is achieved by timing them prior to the beginning of the influenza season to ensure maximum coverage and protection among the population. Likewise, a timely signal to healthcare providers that the influenza season is underway helps to guide their patient management decisions and to mitigate the effects of illness in the individual and in the community.

Local patterns of influenza virus circulation and seasonality may differ geographically, necessitating national estimates of seasonal influenza activity to inform public health guidance. National surveillance data is essential for understanding those patterns and establishing signals for the beginning of the influenza season and epidemic periods. Establishing baseline activity, epidemic and alert thresholds is a useful tool to inform recommendations for timely influenza vaccination to lessen the burden of seasonal epidemics [4].

While several statistical methods are commonly used, there is no gold standard for calculating influenza epidemic thresholds. The methods developed to date vary in their complexity and calculate either time-varying or fixed thresholds. The simplest ones use visual inspection of historical data to create a fixed threshold indicating the expected level of activity throughout the year [5, 6]. Statistical methods include regression models [7–10], time series methods [11], adaptation of industrial control processes such as Shewart charts [12], Cumulative Sum (CuSum) [13] and rate difference models [14].

Methods that involve calculation of means and medians are of medium complexity but are practical as they may be simple to implement. The objective of this study was to evaluate the performance of two methods using means and medians to establish thresholds using data from the Moroccan national influenza-like illness (ILI) syndromic surveillance system. We compare the results of the World Health Organization averages method (WHO method) with the Moving Epidemics Method (MEM) which is recommended by both the WHO and the European Centre for Disease Prevention and Control (ECDC). As a complement to the thresholds using syndromic data, we also calculated a threshold using a composite parameter integrating both syndromic and virologic surveillance data. Following these direct comparisons of the methodologies, we explored the best method for characterizing the 2017/2018 influenza activity.

## Methods

### Data collection

In 2004, the Epidemiology Department of the Ministry of Health of Morocco launched a year-round public sector syndromic surveillance system for ILI comprised of 412 primary health centers, with a catchment population of almost 12 million people. Sites report weekly ILI activity to the regional and central levels, where health officials aggregate the surveillance data. A case definition similar to the 1999 WHO ILI case definition recommended for public health surveillance, defined as “a sudden onset of fever, a temperature >38°C and cough or sore throat in the absence of another diagnosis” was used from 2004 to 2015 [15, 16]. In 2015, Morocco adopted the updated WHO standard ILI case definition [5] developed in 2011 as “an acute respiratory illness with a measured temperature of ≥ 38°C and cough, with onset within the past 10 days” [17]. Reporting includes the total number of ILI consultations aggregated by gender and age group, as well as total outpatient consultations. The proportion of ILI visits among all outpatient consultations is used as a proxy for influenza activity.

In 2007, the Moroccan National Influenza Center (NIC) began a virologic surveillance system in both ambulatory and hospital sites to complement the syndromic system and provide data on laboratory-confirmed influenza activity [18]. After an interruption in data collection beginning in 2010, virologic surveillance was resumed in 8 sentinel sites in 2014. Specimens were collected and characterized between September and June. Enrolling patients from both out- and in-patient facilities allowed the integration of epidemiologic and virologic data representing the spectrum of illness from mild (ILI) to severe (e.g. severe acute respiratory infection or SARI) [17].

We used 11 seasons of syndromic surveillance data (2005/2006 to 2016/2017, excluding the 2009/2010 pandemic year from analysis as influenza activity was not reflective of a typical season); this was described elsewhere [19]. We compared two methodologies for establishing seasonal baseline activity and epidemic thresholds. We also compared the calculated thresholds with the observed weeks for the start and end of the 2017/2018 season. Using three seasons of virologic ILI surveillance data (2014/2015 to 2016/2017), we used the MEM method to make calculations using the composite parameter recommended by WHO [20]; this method estimates the proportion of laboratory-confirmed influenza ILI consultations among all outpatient consultations, or the product of weekly ILI consultations of total outpatient visits and weekly percentage of influenza-positive specimens among respiratory tests.

### Methodology & statistical procedures

#### Overview of WHO and MEM methods

The methods discussed in order to standardize country information on influenza activity, have raised basic concepts summarized in Table 1.

**Table 1:** Summary of WHO method and MEM concepts

Concepts	WHO method (5, 20)	MEM (20, 26)
*Average epidemic curve*	Find 3-week moving average of ILI%. Find median peak week for each season. Align the multiple seasons on median peak week. Calculate the average ILI% for each week. *Indicates the usual level of influenza activity that occurs during a typical year.*	MEM software produces an average curve, lower interval, and higher interval.
*Alert threshold*	Calculate the mean and standard deviation (SD) of the average epidemic curve. For each week, the alert threshold is 1.645 SD above the weekly ILI% mean. ILI% > 1.645 SD indicates high ILI activity or outbreaks and may be used to characterize a severe season.	
*Alert curve*	A graph consisting of the alert thresholds for each epidemic week.	
*Seasonal threshold (WHO) or pre-epidemic threshold (MEM)*	Median weekly ILI% over all weeks (i.e., the average epidemic curve is not used). *Indicates the level of influenza activity that signals the start and end of the annual influenza season(s).*	For prospective surveillance: upper limit of the 95% one-sided confidence interval of the arithmetic mean of the 30 highest pre-epidemic weekly ILI% values. *Parameter value which marks the start of the epidemic period.*
*Post-epidemic threshold (MEM)*		For prospective surveillance: upper limit of the 95% one-sided confidence interval of the arithmetic mean of the 30 highest post-epidemic weekly ILI% values.
*Epidemic period start*	The third of three consecutive weeks with ILI% above seasonal threshold. *Indicates that influenza activity occurs consistently.*	For retrospective analysis of individual season data: see “length of epidemic period”.
*Epidemic period end*	The third of three consecutive weeks with ILI% below seasonal threshold	For retrospective analysis of individual season data: see “length of epidemic period”.
*Length of epidemic period*	Weeks from epidemic start to end.	For retrospective analysis of individual season data: MEM software uses a “maximum accumulated proportions percentage (MAP)” algorithm to split the season into three periods: a pre-epidemic, an epidemic, and a post-epidemic period.
*Epidemic percentage*	Proportion of total cases that occurred during the epidemic period	
*Moderate (WHO) or medium (MEM) intensity*	Upper 40% limit of 1-sided CI of mean of all peak values.	Upper 40% limit of the one-sided confidence interval of the geometric mean of the 30 highest epidemic weekly ILI% values.
*High intensity*	Upper 90% limit of 1-sided CI of mean of all peak values.	Upper 90% limit of the one-sided confidence interval of the geometric mean of the 30 highest epidemic weekly ILI% values.
*Extraordinary (WHO) or very high (MEM) intensity*	Upper 97.5% limit of 1-sided CI of mean of all peak values.	Upper 95% limit of the one-sided confidence interval of the geometric mean of the 30 highest epidemic weekly ILI% values.

#### The WHO method

The 2012 WHO Global Epidemiological Surveillance Standards for Influenza (WHO Manual) [5] included a simple method to establish an average epidemic curve to identify the beginning of the influenza season using national influenza surveillance data. This method characterizes the intensity of influenza activity each year and may be used to describe the seasonality of influenza virus circulation. Using ILI as a proxy for influenza virologic activity [21, 22], we used weekly proportion of ILI among all outpatient consultations as our indicator of influenza activity.

With this method, we were able to produce an average epidemic curve. Using data from the average epidemic curve, we used statistical measures of variance to establish an alert threshold.

**Fig. 1:**
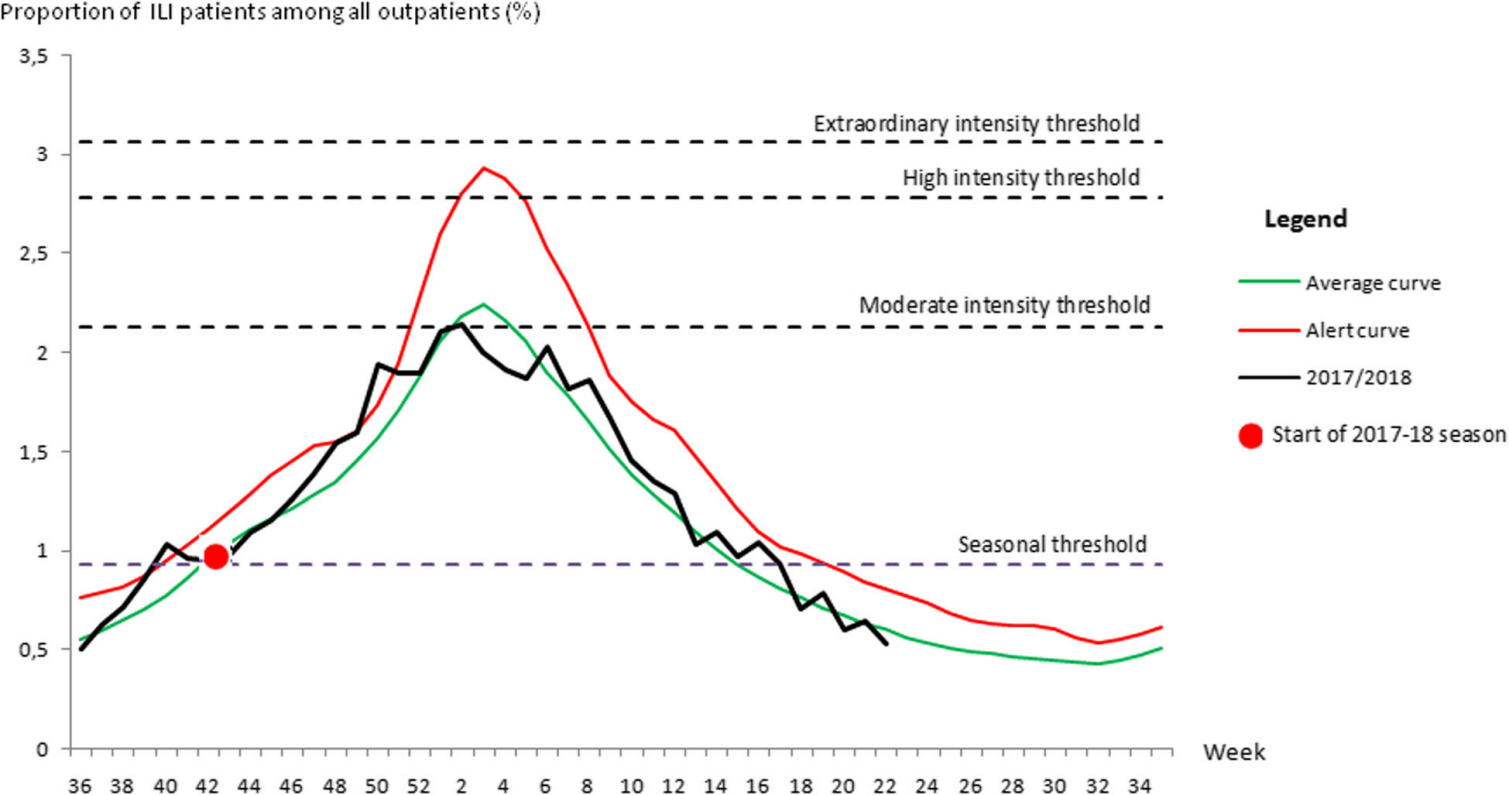
Illustration of the WHO method: plot of the average epedemic curve, seasonal and intensity thresholds based on the weekly proportion of influenza-like illness (ILI) visits all among of outpatient consultations from 2005/2006 to 2016/2017 seasons and observed 2017/2018 season, Morocco

We determined the flat baseline for expected influenza activity throughout the year in order to develop an indicator for the onset of influenza season (seasonal threshold). Sustained influenza activity (i.e., three consecutive weeks) above this baseline indicated the start of the influenza season or the epidemic period [5]. In the final step, moderate, high, and extraordinary intensity thresholds were estimated as described in the WHO Pandemic Influenza Severity Assessment manual [20], (Fig. 1).

#### The moving epidemic method

The Moving Epidemic Method (MEM) [23–28] is an alternative tool developed to help model influenza epidemics also using retrospective national surveillance data. It may be described as a combination rate-difference model that uses cumulative differences in rates to determine epidemic periods and intensity of activity [27, 28].

**Fig. 2:**
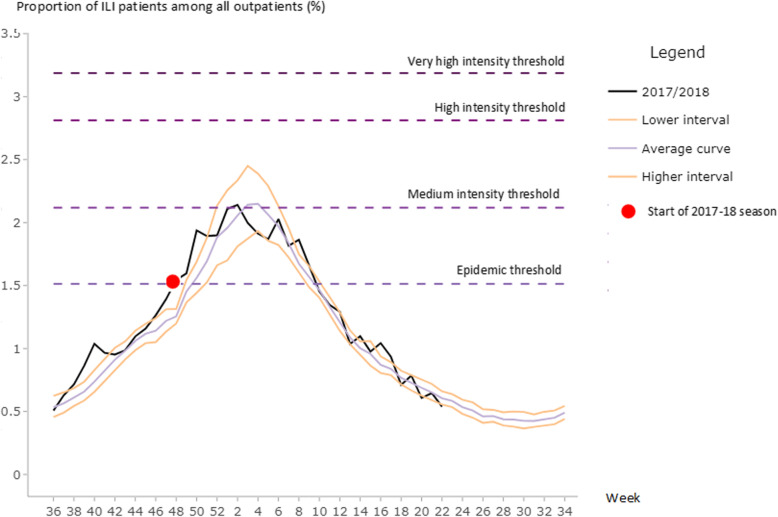
Illustration of the Moving Epidemic Method (MEM): plot of the average epedemic curve, epidemic and intensity thresholds based on the weekly proportion of influenza-like illness (ILI) visits all among of outpatient consultations from 2005/2006 to 2016/2017 seasons and observed 2017/2018 season, Morocco

**Fig. 3:**
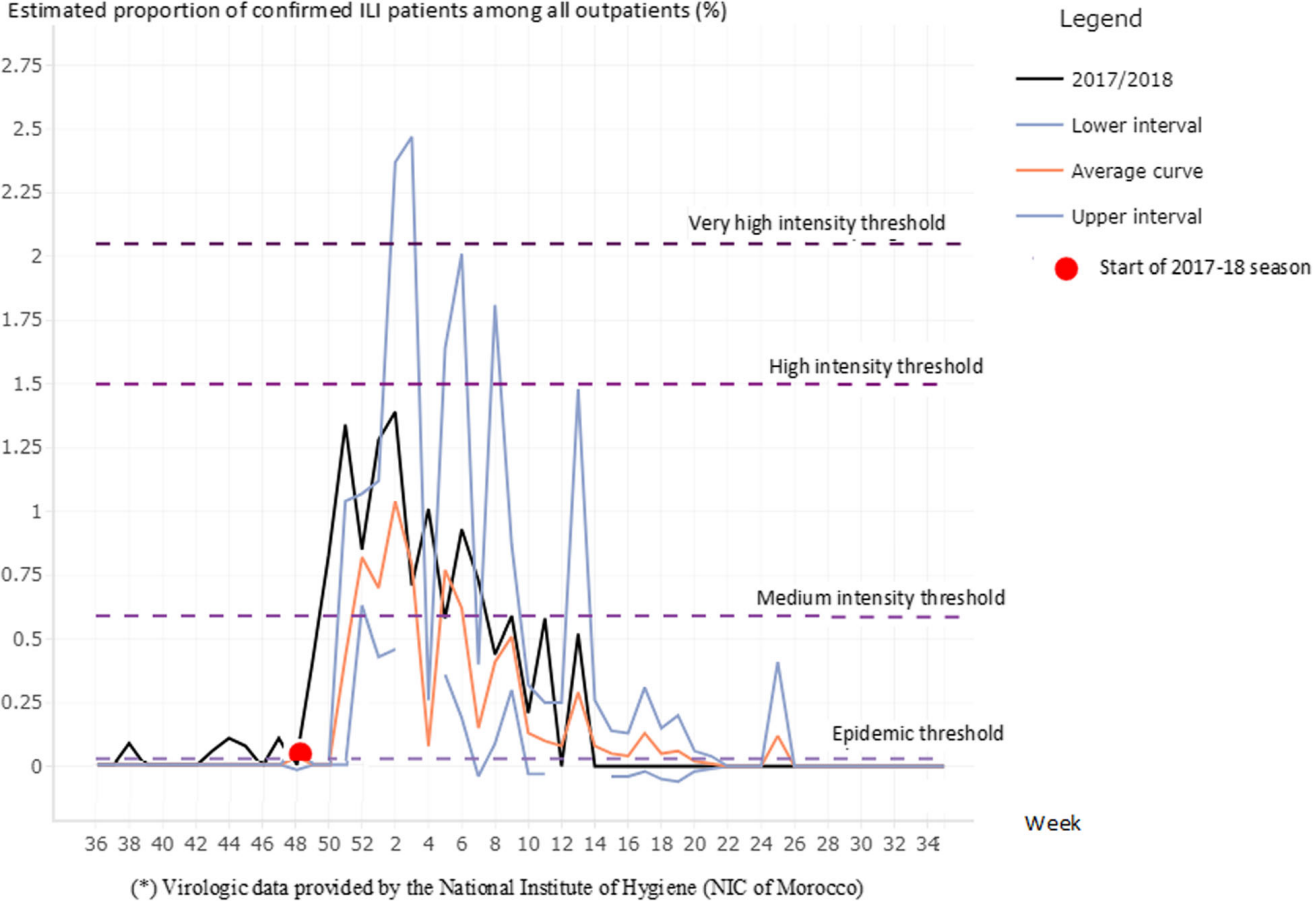
Illustration of the Moving Epidemic Method (MEM): plot of the average epedemic curve, epidemic and intensity thresholds based on composite parameter ^1^ from 2005/2006 to 2016/2017 seasons and observed 2017/2018 season, Morocco

Using the free software R for statistical computing and graphics [25] and its open source user interface RStudio [26], we uploaded our surveillance data via the MEM application [23], and fit the model using three steps. We first visually compared activity over the 11 seasons in order to compare the timing of peak activity and activity trends across seasons. The MEM procedure has three main steps: First, the length, start and the end of the annual epidemics are determined, splitting the season in three periods: a pre-epidemic, an epidemic and a post-epidemic period [27, 28]. In the second step, we built the model by using retrospective data from all 11 seasons. The MEM app calculated the pre-epidemic threshold that marks the start of the epidemic period (analogous to the seasonal threshold in the WHO method). In the third step, medium, high, and very high intensity thresholds were estimated (Table 2). Using the app, we produced graphs of each season showing the pre-epidemic, epidemic and post-epidemic periods (Fig. 2). In addition, as the assumption that ILI activity is reflecting influenza virus circulation has limitations, we created a second seasonal threshold with this methodology using the composite parameter recommended by WHO for three seasons of virologic ILI surveillance (Fig. 3).

**Table 2:** Model estimators using WHO and Moving Epidemics Method (MEM), 2005/2006 to 2016/2017 seasons, Morocco (*)

Estimators used	Analysis method		
	WHO	MEM	MEM
Type of data used	Weekly proportion of ILI patients among all outpatients	Weekly proportion of ILI patients among all outpatients	Estimated weekly proportion of confirmed ILI patients^a^ among all outpatients
Number of seasons analyzed	11	11	3
Average epidemic start week	46^b^	49	50
Average peak week of the seasons 43 to 46	3	3	3
Average epidemic length (in weeks)	24	14	15
Epidemic percentage^c^	38.06%	45.62%	95.41%
Seasonal (WHO) or pre-epidemic threshold (MEM)	0.90%	1.51%	0.03%
**Intensity thresholds**			
Moderate/medium threshold^d^	2.13%	2.12%	0.59%
High threshold	2.77%	2.81%	1.5%
Very high threshold (extraordinary)	3.06%	3.19%	2.05%

(*) 2009/2010 pandemic year excluded

^a^Composite parameter defined as the product of the ILI proportion and the percentage positive

^b^Given the three-consecutive-week-declaration rule considered for the WHO method

^c^Percentage of the cumulative sum of values in the epidemic period of the seasons in the model

^d^Moderate threshold is used for WHO method and medium threshold for MEM

Lastly, we calculated indicators of performance of the app to detect epidemics, using values from the model for sensitivity, specificity, positive predictive value, negative predictive value, percent agreement and the Matthew correlation coefficient (Table 3). The application allowed us to optimize the model by searching the optimum slope of the MAP curve to optimize the goodness-of-fit of the model for detecting epidemics.

**Table 3:** Indicators of the model performance to detect the beginning of an epidemic period (goodness of the Moving Epidemic Method) (MEM) for detecting the epidemics Morocco

Used method Estimators of goodness	MEM	MEM
Historical data	2005/2006 to 2016/2017	2014/2015 to 2016/2017
Type of data used	Influenza Like Illnessproportion (%ILI)	Composite^a^
Sensitivity	0.81	0.76
Specificity	0.92	0.95
Positive predictive value	0.71	0.80
Negative predictive value	0.95	0.93
Percent agreement	0.90	0.91
Matthews correlation coefficient	0.70	0.72

^a^ILI% multiplied by percent of ILI with laboratory-confirmed influenza

The MEM app calculates goodness-of-fit indicators in an iterative process using a cross- validation procedure [27]. True positives (TP) were then defined as values of epidemic period above the threshold, true negatives (TN) as values of the non-epidemic period below the threshold, false positives (FP) as values of the non-epidemic period above the threshold and false negatives (FN) as values of epidemic period below the threshold. The process was repeated for each season in the dataset and all TP, TN, FP and FN were pooled. To measure the performance of the threshold, the following statistics and definitions were used [27]:

1. Sensitivity: The number of epidemic weeks above the pre-epidemic threshold and above the post-epidemic threshold divided by the number of epidemic weeks (epidemic length).
2. Specificity: The number of non-epidemic weeks below the pre-epidemic threshold and below the post-epidemic threshold divided by the number of non-epidemic weeks.
3. Positive predictive value (PPV): The number of epidemic weeks above the threshold divided by the number of weeks above the threshold.
4. Negative predictive value (NPV): The number of non-epidemic weeks below the threshold divided by the number of weeks below the threshold.

#### Ethics statement

The ILI sentinel surveillance system is a public health activity organized by the Ministry of Health of Morocco. Personally identifiable data is excluded from this surveillance system; as a result, no request for authorization from the National Ethics Committees was required. Indeed, the Royal Dahir N°1–15-110 dated August 4, 2015, promulgating the law N°28–13 relating to the protection of persons participating in biomedical research, provides for special provisions for non-interventional or observational researches as stipulated in its articles 2 and 26.

## Results

### Average ILI activity thresholds: WHO methodology

When applying the WHO method to our 11 years of surveillance data, we estimated that the seasonal threshold was the point at which more than 0.9% of outpatient consultations were due to ILI (Table 2). Influenza activity crossed this threshold on average at week 43 and the beginning of the epidemic period would be declared after three consecutive weeks of activity above this threshold, on average at week 46. The typical epidemic period lasted 24 weeks, finishing at week 18, when activity was below the seasonal threshold for three consecutive weeks. The average peak activity occurred during week 3. Seasons where ILI activity regularly crossed the alert threshold may be characterized as severe (Fig. 1 and Table 2). Intensity thresholds were ILI% of 2.13, 2.77 and 3.06% for moderate, high and extraordinary intensity thresholds) (Fig. 1 and Table 2).

### Average ILI activity thresholds: MEM methodology

The MEM model produced an estimate that the average annual influenza epidemic period began on week 49, and that the epidemic period lasted on average 14 weeks. The epidemic threshold (corresponding to the WHO seasonal threshold) was higher, at 1.51% of ILI patients among all outpatients. The average peak activity occurred during week 3, consistent with the estimate using the WHO method. Intensity thresholds were of 2.12, 2.81 and 3.19% of ILI patients among all outpatients for respectively medium, high and very high intensity thresholds (Fig. 2 and Table 2).

Indicators related to the goodness-of-fit of the MEM model for detecting the epidemics, using these retrospective data showed that the sensitivity of the MEM epidemic threshold was 0.81 whereas the specificity was 0.92. Positive predictive value was 0.71 and negative predictive value was 0.95 (Table 3).

### Average laboratory-confirmed influenza activity thresholds: MEM methodology

Using three seasons of virologic data, we established a third seasonal baseline based on the composite parameter recommended by WHO, which integrated both laboratory-confirmed influenza and syndromic ILI reporting (Fig. 3). This method allowed us to compare the results of characterizing seasonality using these data types to identify the beginning of the influenza season. Applying the MEM methodology to our combined data, we determined that the average epidemic began at week 50, average peak activity occurred at week 3 and the average epidemic period lasted 15 weeks. Using this method, medium, high and very high intensity thresholds were set at 0.59, 1.5 and 2.05% of laboratory-confirmed ILI patients among all outpatients (Fig. 3 and Table 2). Goodness-of-fit indicators showed a sensitivity of 76%, specificity of 95%, positive predictive value of 80% and negative predictive value of 93% (Table 3).

### 2017/2018 influenza season performance

2018 ILI data with the WHO/2018 ILI data with the WHO thresholds, the curve overlapped the average epidemic curve and activity crossed the seasonal threshold during week 43 of 2017 and was sustained after this time, confirming that this was the start of the epidemic period (Fig. 1).

The season peaked during the second week of 2018, 1 week earlier than the average identified by the WHO methodology (week 3); we observed peak activity of 2.14% of ILI patients among all outpatients (Fig. 1).

When using the MEM method with ILI proportions, the epidemic period began at week 47, or the end of November 2017. This finding indicated an early season, beginning 2 weeks before the average epidemic start week of 49. The season peaked at week 2 of 2018 (beginning of January), 1 week before the average peak week determined by MEM (week 3), with peak activity above 2% of ILI patients among all outpatients. This season was characterized as one of medium intensity (Fig. 2).

When considering the composite parameter, the MEM method showed that the epidemic period began at week 48, or the end of November 2017, with a sharp increase of the epidemic curve 2 weeks prior to the average start (week 50). The seasonal peak occurred at week 2 of 2018, 1 week before the average peak week (week 3), with peak activity above 1.25% of confirmed ILI patients among all outpatients. This season almost reached the threshold for high intensity (Fig. 3).

## Discussion

The occurrence of the 2009 H1N1 pandemic highlighted the need for a robust and standardized method to make timely assessments of the severity of influenza activity that may be used as an indicator of an unusual event. WHO developed and began implementing a framework on pandemic influenza severity assessment (PISA) [20] in March 2017. Member States are encouraged to establish influenza baseline and epidemic alert thresholds from surveillance data and to monitor and describe the severity of each influenza season (seasonal, epidemic or pandemic influenza) using these thresholds. For this purpose, a simple method proposed by the WHO was used [22, 29, 30]. WHO is now recommending MEM, which is a more sophisticated method of reporting influenza activity adopted by the European Centre for Disease Prevention and Control [31–34] and adopted by several countries from other regions [35, 36]. The analysis using the MEM application with 11 seasons of syndromic surveillance data showed clear seasonality to ILI activity and visual inspection of graphed data revealed a single seasonal peak per year. The data show seasonal peaks between December and March, varying by year, as described by Barakat et al. based on visual analysis [18], matching trends observed in other northern hemisphere countries [37]. The average seasonal peak in Morocco occurs at week 3 (mid-January) using either method. The seasonal threshold established using the method described in the WHO influenza surveillance guidelines was lower than the epidemic threshold calculated by the MEM method when ILI proportions are considered (0.9% versus 1.51% of ILI patients among all outpatients). The average epidemic start week was estimated to be earlier when using the WHO method, with an average start at week 46 versus week 49 or 50 by using respectively ILI proportions or the composite parameter with the MEM method. There is a three- to four-week difference between these 2 methods when describing the typical start to a season; the optimal timing of a seasonal influenza vaccination campaign might vary accordingly. Public health officials must weigh the costs and benefits of the optimal campaign period. Influenza vaccine administration is ideally timed at least several weeks prior to influenza virus circulation as antibody response is achieved on average 2 weeks post vaccination [38]. The average epidemic period estimated by the WHO method was longer compared with the MEM method (24 weeks vs. 14 or 15 weeks respectively). There are few publications with estimates of the typical duration of an influenza season [37]. According to the available evidence, the duration of the influenza season in the temperate zone of the northern hemisphere, ranges 12–19 weeks in Europe [39].

The goodness-of-fit calculations from the MEM application indicate that the MEM capacity for detecting epidemic activity had a sensitivity of 81% and a specificity of 92% when using ILI proportions, implying that it is better for eliminating false signals than it is for detecting a true signal. Our finding is similar to that of Vega et al., who also found the sensitivity to be significantly lower than the specificity [27]. Using Cambodian surveillance data, Ly et al. [30] also found that the WHO methodology appeared to have a higher sensitivity for detecting early epidemic activity, but a lower specificity than MEM, implying a greater risk of signalling false starts to the season. Timely detection of the start of seasonal epidemics may be important to alert health services and to mitigate morbidity, mortality and economic costs by allowing resource allocation and adjusting response measures to face the seasonal overload in the healthcare system. The public health implications for this difference between methodologies are that using the MEM method without applying the seasonal threshold established using the WHO method, there is a risk of missing the beginning of the epidemic period and not providing timely guidance to clinicians to indicate influenza season has begun, and to manage patient treatment accordingly. Using the lower WHO threshold for public health messaging regarding the beginning of the influenza season may pose the risk of a false alert and perhaps over-prescribing antiviral medications. From another point of view, using a low seasonal threshold could influence decision-makers to recommend earlier vaccination. As our results showed that the seasonal threshold typically occurs between mid-November and mid-December in Morocco, appropriate timing for vaccination could be about 1 month before this date. Of note, the US Advisory Committee on Immunization Practices (ACIP) recommends that vaccination should be offered by the end of October, considering the unpredictability of timing of onset of the influenza season and concerns that vaccine-induced immunity might wane over the course of a season [40].

Low seasonal thresholds may be crossed multiple times as was the case in our application of the WHO threshold for several seasons (2005/2006, 2006/2007, 2010/2011, 2011/2012 and 2013/2014 [not shown]), due perhaps to variability in reporting by the surveillance sites. Because of this variability, it is possible that declaring the start of the influenza season after two or three sustained weeks of activity above the threshold as recommended by WHO, is a prudent option for considering influenza transmission as epidemic. The MEM methodology, however, calculates the length of the epidemic period during each season separately in order to determine the average length. Thus, the epidemic threshold calculated with the MEM method could be preferable to that established with the WHO method.

MEM was first used in in the WHO European Region to estimate epidemic period and intensity using a minimum of five historical seasons for the calculations and the target season [27]. Despite the availability of only 3 years of virologic data in Morocco, we followed a WHO recommendation to use the composite parameter with MEM [20]. This allowed a clearer cut estimation of the beginning of the influenza epidemic period, characterized by a sharp increase in influenza-confirmed ILI cases.

When ILI proportions are used, the two methods produce similar values for each intensity threshold considered in the PISA assessment of seasonal transmissibility; WHO has adopted the MEM for this purpose. When comparing the highest weekly activity per season (the seasonal peak) to the intensity thresholds established by WHO and MEM procedures, the 2017/2018 season was of moderate intensity (Figs. 1 and 2). Using the composite parameter, the 2017/2018 seasonal peak nearly reached the high intensity threshold, whereas this curve didnot cross the medium intensity threshold when using only ILI proportions.

Our study has several limitations. First, the assumption that ILI activity reflects influenza virus circulation is limited because of possible concurrent circulation of other respiratory viruses (e.g., RSV) [41, 42]. WHO recommends using a composite parameter defined as the product of the ILI or ARI proportion and the percentage positive for the transmissibility indicator of the PISA tools [20]. Unfortunately, virologic data collected prior to 2014 was not consistently available for the period of our study as virologic surveillance was disrupted between 2010 and 2014. Despite this limitation, our laboratory-confirmed data showed something different than the syndromic data as the start of the virologic activity occurs suddenly and is therefore clearly identified. It is obvious that the inclusion of virologic data increases the specificity of seasonal threshold estimation. According to the WHO guidelines [5], *“a combination of parameters may be preferable. For example, a seasonal threshold could be defined as the week in which the ILI rate crosses a certain value and the percentage of specimens testing positive reaches a certain point”*.

Given the long life of our surveillance system, our data were limited by changes in data collection practices, inconsistency of reporting by surveillance sites, and variable access to primary health care. These problems are not unique to the Morocco ILI surveillance system, and we believe they are the nature of routine, sentinel surveillance. Another limitation was the adoption of a new case definition in 2015, at which point we also re-launched our surveillance system using a new protocol. These changes may have affected the trends that we observed in ILI activity from that year forward. Since both methods we used to establish thresholds recommend using a minimum of three to five seasons of data, we would not have enough data to run the models if we used only data from 2015 onward.

Determining a gold standard for influenza epidemic and intensity thresholds has been a long-standing research question for both international organizations and country-level public health authorities, and there is no consensus on the best method [5, 27, 28, 37, 43–45]. Both the WHO method and the Moving Epidemic Method translate quantitative trend data into standardized qualitative intensity levels, which permit countries to determine if the current season is atypical or to assess country or regional differences in activity and intensity. Both methods identified that the 2006/2007 season was the most active in Morocco, excluding the 2009/2010 pandemic season according to non-published observations. Both methods are coherent to identify excess activity or high intensity thresholds even though with adequate laboratory data MEM with the use of the composite parameter, gives a theoretically better qualitative measure of the level of activity.

## Conclusions

This comparative study has shown that the threshold methodology presented in the WHO manual is simple to implement and easy to adopt for use by the influenza surveillance system in Morocco or the national surveillance systems of other similar countries. MEM is more statistically sophisticated and may provide a more accurate detection of the start of seasonal epidemics in temperate countries with clear seasonal circulation of influenza viruses, especially if virologic data are considered. Whichever method is used, analysis of surveillance data will provide information about seasonal thresholds and epidemic curves that may help health care personnel in the clinical management of respiratory illness after the start of influenza season. Establishing a seasonal threshold for influenza helps health authorities to identify suitable periods for annual vaccination campaigns and for health practitioners to administer influenza vaccines or prescribe influenza antiviral drugs. Computerization of the influenza surveillance system improves timeliness and assessment of the intensity of the influenza epidemic early in its course will guide policymakers in ensuring the appropriate allocation of resources to control seasonal epidemics.

## Data Availability

Datasets were collected by each participating site including the National Influenza Center and gathered on a pooled database at the Direction of Epidemiology and Disease Control of the Ministry of Health of Morocco. Data cannot be publicly shared due to internal regulations of the Ministry of Health of Morocco. The datasets analyzed during the current study could be available from the corresponding author on reasonable request and with special authorization of the Ministry of Health of Morocco.

## Abbreviations

ACIP: US Advisory Committee on Immunization Practices
ARI: Acute respiratory infection
CI: Confidence interval
ECDC: European Centre for Disease Prevention and Control
FN: False negative
FP: False positive
ILI: Influenza like illness
ILI%: Proportion of ILI visits among all outpatient consultations
MAP: Maximum accumulated proportions percentage
MEM: Moving epidemic method
NIC: National Influenza Center
NPV: Negative predictive value
PISA: Pandemic Influenza Severity Assessment
PPV: Positive predictive value
RSV: Respiratory Syncytial Virus
SARI: Severe acute respiratory infection
SD: Standard deviation
TN: True negative
TP: True positive
US CDC: US Centers for Disease Control and Prevention
WHO: World Health Organization
